# Supplementary material for: EnzML: multi-label prediction of enzyme classes using InterPro signatures
Source: BMC Bioinformatics. 2012 Apr 25;13:61. doi: 10.1186/1471-2105-13-61 (PMC3483700; doi:10.1186/1471-2105-13-61)
Supplement: Addtional file 5 — The Java code to format the data files, evaluate and predict. The file enzml_java_code.tar.gz contains the Java code used to format database data to ARFF and XML formats, to execute cross and train-test (jackknife) evaluations and to record evaluation results to database. More information is included in the readme.txt file and the Javadoc files. The code can be used with a MySQL database. To use a different database software, other JDBC drivers might be required. [file 1471-2105-13-61-S5.gz › java_code/enzml2011/doc/uk/ac/ed/inf/taweka/mulan/xml/package-use.html]

Uses of Package uk.ac.ed.inf.taweka.mulan.xml


---


|  |  |  |
| --- | --- | --- |
| **Overview** \| **Package** \| Class \| **Use** \| **Tree** \| **Deprecated** \| **Index** \| **Help** | |  |
| PREV   NEXT | **FRAMES**    **NO FRAMES**     **All Classes** |  |


---


## **Uses of Package uk.ac.ed.inf.taweka.mulan.xml**

| Packages that use uk.ac.ed.inf.taweka.mulan.xml | |
| --- | --- |
| **uk.ac.ed.inf.taweka.mulan.xml** |  |
| **uk.ac.ed.inf.taweka.test.mulan.xml** |  |

Classes in uk.ac.ed.inf.taweka.mulan.xml used by uk.ac.ed.inf.taweka.mulan.xml

****MulanLabel****
  
          A node in the Mulan XML (a label for machine learning) \*

Classes in uk.ac.ed.inf.taweka.mulan.xml used by uk.ac.ed.inf.taweka.test.mulan.xml

****MulanLabel****
  
          A node in the Mulan XML (a label for machine learning) \*

****MulanXml****
  
          Generates an XML file for labels in the Mulan format
http://mulan.sourceforge.net/ http://mlkd.csd.auth.gr/multilabel.html

---


|  |  |  |
| --- | --- | --- |
| **Overview** \| **Package** \| Class \| **Use** \| **Tree** \| **Deprecated** \| **Index** \| **Help** | |  |
| PREV   NEXT | **FRAMES**    **NO FRAMES**     **All Classes** |  |


---
